# Inferring human behavior through online social networks may provide accurate behavioral estimates for outbreak forecasting of arboviruses

**DOI:** 10.1371/journal.pgph.0004889

**Published:** 2025-07-24

**Authors:** Frédéric Jourdain, Debapriyo Chakraborty, Beatrice Gaillard, Arnaud Gautier, Frédéric Simard, Pierre Jay Robert, Laurent Dormont, Jean-Claude Desenclos, Benjamin Roche

**Affiliations:** 1 Santé Publique France, Saint-Maurice, France; 2 MIVEGEC, University of Montpellier, CNRS, IRD, Montpellier, France; 3 CEFE, University of Paul-Valéry Montpellier 3, University of Montpellier, CNRS, EPHE, IRD, Montpellier, France; New York University Grossman School of Medicine, UNITED STATES OF AMERICA

## Abstract

Human behavior is known to be a fundamental, yet often neglected, component of infectious disease epidemiology, especially during outbreaks. To quantify its role and fluctuations, analyzing message contents on popular online social networks – part of so-called digital epidemiology – is a promising approach. However, such methods could be biased and generate estimation errors since social media users may not be representative of the general population. To address this, we systematically compared social media-derived estimates with those obtained from a large-scale opinion survey. In the setting of metropolitan France, where the risk of arbovirus outbreaks is increasingly important, we compared the frequency of three types of emotional states related to human-mosquito contact identified in 160,000 messages on X (formerly Twitter) with the frequency of the same emotional states expressed through a large-scale opinion survey involving 15,000 people during the same period. Both sources of data were used to parametrize a mathematical model of mosquito-borne virus transmission. We found that estimates of these emotional states for different age groups in the opinion survey could be highly different from estimates based on X data. Nevertheless, by integrating demographic adjustments and incorporating variability into our transmission models, we showed that the predicted overall outbreak dynamics remain comparable under certain conditions. This study provides the first evidence that using digital social network data to infer epidemiologically relevant behavior achieves similar results as using large-scale opinion survey data. These outcomes highlight that X data could be used to help forecast outbreaks dynamics, opening new opportunities for real-time assessment of human health-related behavior and the definition of control strategies.

## Introduction

Today, human behavior is recognized as an important component of infectious disease transmission dynamics [1–3]. Examples include, but are not limited to, the impact of sexual behavior on sexually transmitted infections [4], burying rituals during Ebola outbreaks [5], vaccine refusal and its impact on childhood diseases [6], water storage [7], health care seeking [8] or disease perception [9] affecting vector-borne diseases, and many others. Despite the recognized importance of behavior on the emergence and transmission of infectious diseases, it is still often neglected [10], especially in the context of outbreak response, even though public health interventions [3,11] and mathematical models [12] have demonstrated that human behavior can dramatically change the dynamics of an outbreak.

Measuring health-related behavior is challenging since behavior may change over time and place which may partially explain the lack of behavioral parameters in mathematical models of disease dynamics [13]. Health-related behaviors are driven by cognitive and emotional processes [14]. Emotional states and risk perceptions are known to influence behavior positively or negatively [15] and can therefore be used as proxies for some health-related behaviors. To do so, public health agencies generally rely on large-scale opinion surveys, most of which were inspired by the Behavior Risk Factor Surveillance System (BRFSS), a large survey implemented each year since 1984 in each state of the United States of America [16]. Such behavioral studies are widely implemented to assess the level of awareness on a specific topic as well as the practices of a target population before planning and implementing prevention programs. However, such surveys are expensive and time-consuming to set up, and cannot respond to concerns that arise in an emergency context. They further require the enrolment of a large random sample of the population.

To overcome these difficulties, online social network data is a promising alternative tool [17]. Textual analyses of messages exchanged on online social networks can be used to explore disease activity [18], develop early-warning systems [19,20] or assess human behavior given a certain threat [21,22]. The enormous amount of immediately available data opens promising opportunities to monitor in real-time how populations react to disease activity in terms of behavior.

However, it is well known that online social networks can be demographically biased. For example, in the UK a study showed that X users were younger and more often male compared to the general population [23]. Therefore, the relevance of such data to provide accurate and real-time indicators of population behavior for modelling and predicting infectious disease transmission has yet to be demonstrated to unlock its full potential for public health management authorities.

In this study, we tackled this issue with the case of potential arbovirus outbreaks in Metropolitan France, an area where this risk is continuously increasing following the progressive invasion of *Aedes albopictus* over the last 15 years [24]. We compared the frequency of three emotional states or attitudes that are expected to influence behaviors involved in the occurrence of contacts between human and mosquito populations expressed in social media, with those captured by a large-scale statistically representative opinion survey. We then integrated these two ways of assessing behavior of the population into mathematical models to see if the expected dynamics of arbovirus outbreaks are significantly different depending on whether the model has been parameterized with X rather than opinion survey data.

## Materials and methods

### Ethics statement

We used data from the “French Health Barometer 2016” (and hereafter called “Barometer”). The Health barometers are national telephone surveys, implemented since 1992, which aim to monitor over time the main behaviors, attitudes and perceptions related to health risks and the state of health of the French population with regard to smoking, alcohol and drug consumption, vaccination practices, sexual behavior, physical activity, nutrition, quality of life, sleep, accidents, mental health, etc. It provides a representative snapshot of the French population, supporting public health actions by informing, adapting, and evaluating prevention strategies. Stratification is based on age, gender, and geographic region. It has received official approvals for statistical quality, general interest, and ethical compliance from National Council for Statistical Information (CNIS) and the Expert Committee for Research, Studies, and Evaluations in the Health Field (CESREES). Participation in the survey is mandatory to ensure comprehensive and reliable data. Further details on the methodology can be availed from barometre-spf.fr.

### Behavior and emotional states considered

In this study, we focused on the risk of arbovirus outbreaks in metropolitan France. We considered three different emotional states or attitudes that are likely to be linked to behaviors influencing the intensity of pathogen transmission, either positively or negatively [3,12,25]. These emotional states or attitudes were also selected as they are expressed sufficiently frequently to be quantified both on X and in large-scale opinion surveys.

Nuisance indicates the discomfort caused by mosquitoes, which can signal their local abundance due to the study population’s lack of habituation to their presence. This emotional state is particularly relevant in mainland France, where the invasive mosquito species Aedes albopictus has been spreading since 2004 and potentially remains novel to many inhabitants [26,27]. High nuisance levels may motivate behaviours such as the use of repellents or improved vector control measures, thereby potentially reducing transmission. Conversely, heightened nuisance could also indicate elevated mosquito density, which would increase human-vector contact and thus transmission risk.

Fear of arboviruses is also expected to increase risk perception [28], especially as the very plausibility of the risk in France is reinforced by the occurrence of previous events of autochthonous transmission [29]. Fear can promote protective behaviours such as avoiding outdoor activities at peak mosquito times or adopting personal protective measures like insecticides or window screens. However, excessive fear could lead to panic-driven behaviours or misinformation, potentially complicating public health interventions.

The curiosity for control methods is expected to be linked to the fear of arboviruses and the local abundance of mosquitoes [26,30,31]. This emotional state may represent an active search for solutions and knowledge to mitigate risks. Individuals displaying curiosity might adopt preventive behaviours, such as researching and implementing mosquito control measures (e.g., eliminating breeding sites), which could reduce transmission intensity.

Each of these three emotional states (hereafter called ‘Nuisance’, ‘Fear’ and ‘Control’ respectively) could be linked to the desire to protect oneself, which likely reduces the risk of transmission. However, they can also signal high mosquito abundance, which are important drivers of virus transmission. Understanding the balance of these effects is critical for predicting their overall impact on disease dynamics.

### X *analysis*

*Using the X API and repeated requests, we* downloaded all tweet messages on the X platform, including the word mosquito in French (“*moustique*”), from the year 2016. Then, we classified those tweets in a semi-automatic way with the NVivo software. We identified all words included in tweets with more than four letters and that were present in at least 100 tweets to ensure statistical reliability. Two independent analysts classified each word into one of the three emotional states (‘Nuisance,’ ‘Fear,’ or ‘Control’). When disagreements arose, they were resolved by consensus. Words unrelated to these three emotional states, even if mosquito-related, were excluded from the analysis. This ensured that only the most relevant words were included in our analysis. Finally, we computed the minimum and maximum frequencies for each emotional state across the different classification-based scenarios. It was possible that given X message could be classified more than once if it included different words associated with different emotional states. All data collection and analysis were conducted in compliance with the terms and conditions of the X API and adhered to ethical guidelines. No personally identifiable information was collected or analysed.

### Large-scale random telephone survey for behavior risk factors in the French population

In 2016, 14 questions on vector-borne pathogens (see S1 File) were added to the Barometer to allow a comparison with the analysis of X’s content. The Barometer survey uses a two-stage stratified sample based on the random generation of landline and cellular phone numbers. At least 40 attempts were made to complete an interview for every sampled number if no response. The calls were made at different times of the day, and on different days of the week to maximize the chance of contact with a potential respondent [32]. In the Barometer survey, the participation rate was estimated at 50%, and 15, 216 individuals between 15 and 75 years living in mainland France were interviewed. For each question, different levels of answers, representing the intensity of the emotional states, were proposed (e.g., not afraid, slightly afraid, afraid or extremely afraid). To quantify the variability of the expression of each emotional state within each age class, we considered three different thresholds of answer intensities (e.g., at least slightly afraid, at least afraid, and extremely afraid only). Then, we computed the minimal and maximal frequency of each emotional state across these three different thresholds of answer intensities for each age class.

The collection and analysis of these datasets complied with the terms and conditions for the source of the data.

### Mathematical model

We designed a simple mathematical model stratified by age that included the influence of emotional states from each of the possible data sources. The deterministic form of this model is as follows (simulation has been made stochastically using the Direct Gillespie method [33]):


$$\frac{dS_{i}}{dt}=-\beta_{i}S_{i}\sum_{i}^{n}I_{i}$$

$$\frac{dI_{i}}{dt}=\beta_{i}S_{i}\sum_{i}^{n}I_{i}-\sigma I_{i}$$

$$\frac{dR_{i}}{dt}=\sigma I_{i}$$

Relying on a classic Susceptible-Infectious-Recovered (SIR) framework, the whole population is divided into *n* different age groups, represented by the index *i*, and different infection status. Starting being Susceptible (S), individuals become infectious (I) through a density-dependent transmission rate *β*_*i*_, and then recovered (R) through recovery rate *σ*. Because we simulate short periods of time (only 6 months, representing the amplification phase of an outbreak), we neither considered changes in human demography nor in the mosquito population dynamics. Transmission rate is impacted by human behavior as follows:


$$\beta_{i}=\beta_{0}(1+\sum_{j}\gamma_{ij}\delta_{ij})$$

where *β*_*0*_ is the basal transmission rate, *j* is the emotional state considered (j = ’Nuisance’, ‘Fear’ or ‘Control’), γ its contribution for transmission to age class *i*, and δ its frequency within the age class (as inferred by our data sources). Our model assumes that human behavior, as inferred by our data sources, are impacting only the probability to become infected by a mosquito (i.e., transmission rates are identical between every age class as well as within each age class)

### Simulation and analysis

We simulated separately the different impacts of each emotional state on the transmission rate *β*_*i*_, with a magnitude $\gamma_{ij}$ ranging from -0.7 to 0.7, within a population of 100,000 inhabitants, across two situations where data from X and data from the Barometer were considered separately. The parameter values capture both protective behaviours (negative gamma) and behaviours that increase contact rates (positive gamma) assuming arbitrarily that the maximum contribution is unlikely to be more than 70% of basal contact rate. It is worth mentioning that a gamma value that equals zero would represent outbreak dynamics without any impact of human behavior. Since we did not have age information of X users, our model investigating X data assumed a homogenous population with only one age class (i = 1), and the emotional state frequency estimated through X. Conversely, our model including Barometer data considered nine different age groups, and the frequency of each emotional state measured. We included the age distribution of the French population (Source: the national statistical institute, Paris https://www.ined.fr/fr/tout-savoir-population/chiffres/france/structure-population/pyramide-ages/) to reflect the contribution of each age group.

We selected basic parameters values ($\beta_{0}$ and σ) in order to generate a system with an average basic reproductive number of 2, which is consistent with what is expected for arbovirus outbreaks in Metropolitan France (Liu et al., 2020; Sochacki et al., 2016), and we ran 1,000 replicates for each model (with a unique combination of one emotional state, one magnitude and one data source). Then, we recorded the distribution of the numbers of infectious individuals at the peak of the outbreak, as well as the distribution of the number of weeks to reach the peak. To compare the forecasted epidemiological outcomes of an outbreak relying on the Barometer poll or X data, we computed (1) the difference between the time to reach the epidemic peak according to both data sources, and (2) the difference between the proportions of infectious individuals at the epidemic peak according to both data sources.

Ethics statement: No formal consent from participants was obtained due to anonymous data

## Results

**Fig 1:**
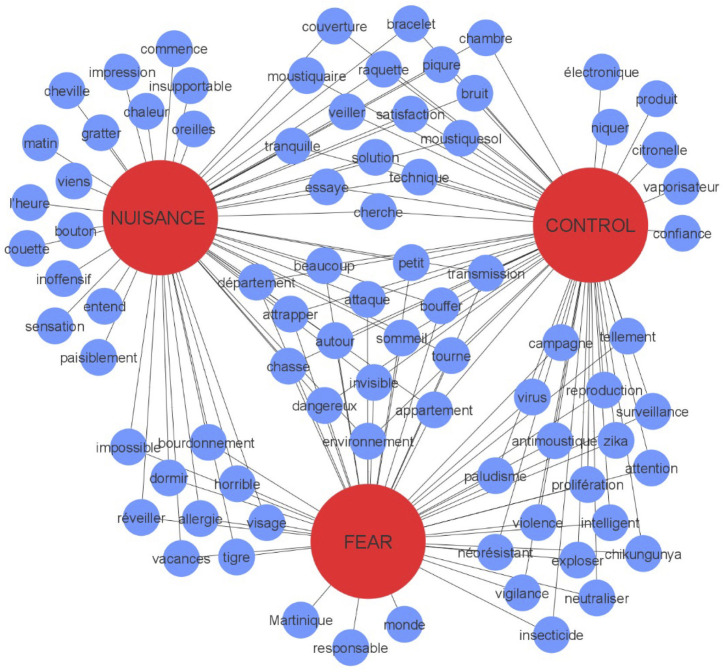
Relationship between the three emotional states or attitudes investigated, and the most common words identified in social media content (X, formerly Twitter).

On October 18^th^ 2018, 163,384 messages (tweets) posted in 2016 on the social media platform Twitter (now called X) with the word “moustique” (mosquito in French) were retrieved. Across these tweets, 82 relevant words for tweet classification were identified (S2 File). These words were the most frequent ones that are not connecting words (such as “and”), pronouns (such as “we”) or verbs. 37 were linked with the emotional state “Nuisance”, 24 with the state “Control” and 21 with the state “Fear”. Among them, 42 words had an ambiguous meaning with a second emotional state and 15 with a third one. Fig 1 shows how the tweets including these words have been classified, which allows inferring a confidence interval on the frequency of each of the three emotional states considered. The figure reveals the variability in word classification, with some words uniquely linked to one emotional state, while others are associated with multiple states, indicating overlaps in emotional expression. In total, 255,001 classifications have been made over all tweets, representing a robust dataset for analysing emotional states in mosquito-related tweets.

**Fig 2:**
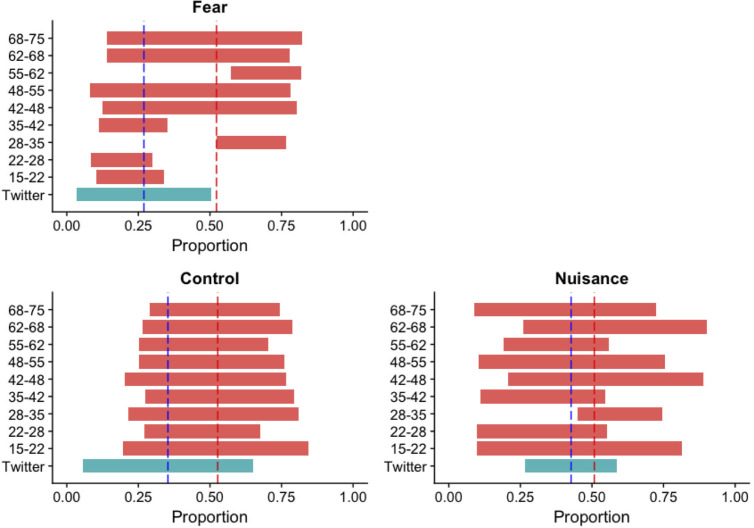
Comparison of emotional states frequencies estimated from the Barometer with those estimated from tweets. The green bars represent the variability in emotional state expression estimated on X, and the red bars represent the variability in feeling expression estimated for each age group in the Barometer. See main text for details on variability quantification.

In Fig 2, we compare the estimates of the frequency of each emotional state in X data with those quantified on the different age classes in the Barometer survey, summarising the difference between the two datasets. While the frequency of “Control” state is homogeneous across age groups in the barometer dataset, its mean is significantly greater than the estimate from the X data (p-value < 0.05). Conversely, frequencies of states “Fear” and “Nuisance” are heterogeneous across age groups in barometer data, and their mean is only significantly different for the state “Fear” (p-value < 0.05). Therefore, only the “Nuisance” emotional state shows a similar mean frequency between the Barometer poll and X data (p-value > 0.05).

**Fig 3:**
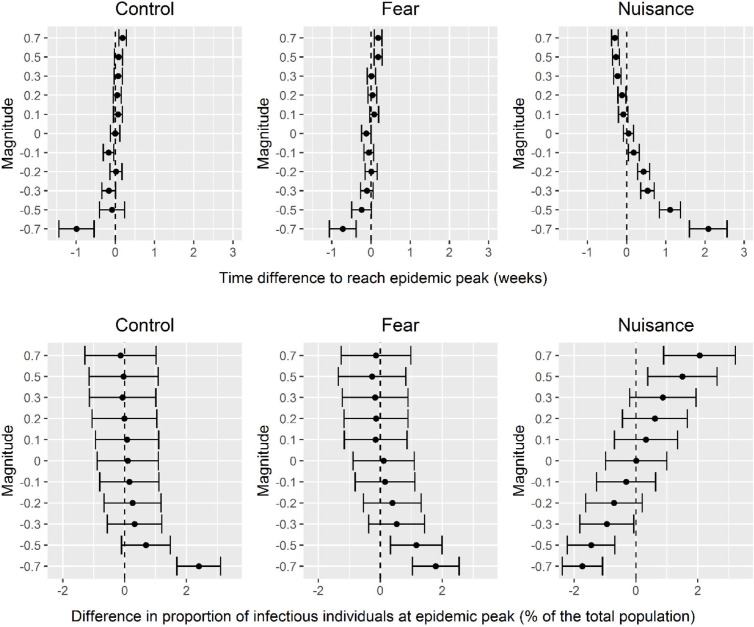
Differences of impact on pathogen transmission rate predicted by our model when considering emotional states data coming from X (all ages aggregated), and multiple age groups and emotional state data coming from the Barometer. Above: distribution of differences in time to reach the epidemic peak, expressed in number of weeks. Below: distribution of differences between the proportions of infected individuals, expressed as a percentage of the total population.

Despite these differences in the estimates of emotional state frequency between X and the Barometer, the predicted outbreak patterns from both sources were similar from a public health perspective (Fig 3). The difference in the expected time of the epidemic peak was less than one week for any of the three emotional states, regardless of their impact on transmission rate. An exception was the “Nuisance” state, where larger impacts on transmission (-0.7 and -0.5 magnitudes) resulted in he epidemic peak occurring 2 weeks and 1 week earlier, respectively. the difference of 2 weeks and 1 week respectively. The difference in the proportion of infectious individuals at the epidemic peak remains limited for both data sources, typically less than 1% and almost always less than 2%, highlighting that social network data can be as reliable as broad opinion surveys for outbreak predictions.

## Discussion

Public health policies are largely built on population-based surveys to assess the level of awareness on a specific topic, as well as the practices of a target population before planning and implementing prevention programs. However, such surveys have difficulties to adequately address the evolution of risk perception and practices that are particularly rapid and significant in the context of an emerging situation. In this study, we addressed this gap by using data from an online social network to parametrize a mathematical model, testing its application in forecasting arbovirus outbreaks in metropolitan France. Our study aligned with previous studies that attempted to integrate human behavior into infectious diseases models [2,12,34].

We have shown that the frequency of emotional states expressed on X could constitute a source of data for the integration of the impact of human behavior on the dynamics of vector-borne diseases in metropolitan France. While the emotional states estimated through X and the Barometer differed significantly through the age pyramid, the overall outbreak dynamics predicted by classic mathematical models remained very similar for both data sources. The difference between the observed delays is undoubtedly the most important one, as this difference directly reflects the advantage of using data from social media in forecasting and therefore in decision support. However, a delay of one week is perfectly representative of the response delays observed during events of autochthonous transmission in temperate areas, whether in terms of active search for secondary cases, or the implementation of vector control operations [35].

Several reasons may be mentioned to explain the differences in frequencies of emotional states estimated from X (where younger people likely dominate) and the Barometer survey. These are more important than the difference in their epidemiological consequences when these frequencies are integrated into mathematical models. The first reason is that considering the real age pyramid tends to decrease the importance of these differences by allocating to each age group a contribution proportional to its weight within the whole population, which can offset the impact of the differences observed in emotional states frequency.

Second, it appears that the emotional state “Control” gives the best match between theoretical expectations based on different data sources because there is much less heterogeneity across age groups even if the mean frequency between the two data sources remains quite different. This result opens interesting perspectives by suggesting that the most relevant emotional states measured on X are those with the lowest heterogeneity by age. Therefore, our work shows that it would be possible to rely on population-based surveys to identify emotional states or perceptions to follow on social networks, to benefit from this type of data in near-real time in a “now-casting” perspective.

From an epidemiological perspective, the good match between our models relying on X and Barometer when considering the attitude of “Control” echoes to previous studies that have already linked this emotional state with arbovirus transmission intensity during an outbreak setting [21] or preventive behavior during an inter-epidemic period [31]. While these previous studies took place in a tropical environment, our results from mainland France suggest that this approach of inferring human behavior may also be relevant across environments, broadening the environmental, sociological and epidemiological scope of its potential use.

Several choices we made deserve more explanation. Firstly, the selection of the three emotional states may be questioned in the context of their relevance to protective behaviors. While other emotional states could have been considered, the goal of this study was to show the relevance of estimating these emotional states for outbreak forecasting. Other emotional states could indeed have been considered. However, it was necessary to retain emotional states for which there was evidence in the scientific literature of causal inference between the expression of these emotional states and protective behavior regarding host-vector contact. Moreover, it was also necessary for the expression of these emotional states to be frequent enough to be quantified. However, it remains clear that other attitudes, perceptions or emotional states could have been considered. Therefore, the critical point was to estimate the frequencies of the expression of contrasted emotional states to compare outbreak dynamics using population-based survey and X data, and not to identify the most relevant emotional state to achieve this goal. To do so, specifically designed comparative studies involving different emotional states would be needed.

In the behavioural analysis step of our methods, we prioritised word meaning accuracy and epidemiological interpretability, which required detailed contextual understanding of the tweets. Manual annotation allowed experienced epidemiologists to carefully resolve ambiguities in word meanings, creating a reliable, epidemiologically meaningful classification. However, the manual annotation is time-consuming, labour-intensive, and impractical for operationalisation, which will require real-time analysis of massive social media datasets in French. In this context, the manual approach was deliberately chosen to establish a high-quality proof-of-concept necessary for exploring future automated methods. As a next step therefore, it is possible to train and validate French-language (or any other language) ML models based on an expanded annotated dataset to support future automation of our digital epidemiological framework.

We could have used more complex models to investigate the epidemiological consequences of the different methods of emotional state inference. However, within our context of arbovirus outbreaks in metropolitan France, greater complexity is not likely to dramatically affect the outbreak dynamics over the first 6 months. Therefore, while further work is needed to turn this approach into a translational decision-making tool for outbreak control and prevention, our results indicate that the heterogeneity of emotional states is likely important for outbreak forecasting. Tracking changes in human behavior in real-time and the anticipation of their epidemiological consequences are crucial levers for outbreak control. To this extent, the amount of data provided by online social networks is a valuable and useful opportunity to quantify the otherwise complex behavioral component of pathogen transmission dynamics. This study, for the first time, provides evidence on the relevance of this tool for outbreak forecasting. Further multidisciplinary research is needed to identify and quantify more precisely the most important and relevant emotional states to track, paving the way for its translation as an operational tool for outbreak control and prevention.

Another limitation of the study is that it focuses solely on mosquito-borne arboviruses, and the findings may not generalise to other vectors like ticks with different ecology and host-seeking behaviour than mosquitoes. For instance, Lyme disease (a tick-borne disease) in rural areas is often driven by outdoor recreational behaviours and seasonal changes, perhaps requiring different behavioural insights into people. Validating our framework for non-mosquito vectors will be a key step for broader applicability.

The policy implications of this work are significant in regard to the role of digital epidemiology in outbreak forecasting and preparedness. While real-time sentiment data from social media platforms can increase our situational awareness regarding epidemic risk, our findings highlight the importance of demographic adjustment and transparent methodological frameworks. Without these guardrails, social media signals may distort perceptions of population-level behaviour.

Integrating real-time data from social media offers a promising avenue for accelerating outbreak forecasting, and therefore faster and equitable response. During an outbreak, social media data could provide immediate feedback on public sentiments (e.g., increased “nuisance” or “fear” expressions), enabling rapid prioritisation of interventions like insecticide spraying. Simultaneously, surveys could ensure that strategies address demographic nuances and offline and underrepresented populations, such as targeted interventions (for example age-based priority in vaccination) and communication for elderly populations.

To operationalize this approach, we recommend that public health authorities invest in integrated data platforms, develop standardized protocols for correcting demographic biases, and implement robust data governance mechanisms that uphold privacy and ethical use of behavioural data.

## Supporting information

S1 File Questions used within the “Barometer of Health”. (DOCX)

S2 File Words identified with Nvivo. (DOCX)
