# Supplementary material for: Inferring human behavior through online social networks may provide accurate behavioral estimates for outbreak forecasting of arboviruses
Source: PLOS Glob Public Health. 2025 Jul 24;5(7):e0004889. doi: 10.1371/journal.pgph.0004889 (PMC12289027; doi:10.1371/journal.pgph.0004889)
Supplement: S1 File — (DOCX) [file pgph.0004889.s001.docx]

***Questions used within the “Barometer of Health”***

Concernant la présence de moustiques chez vous et autour de chez vous, hors période hivernale, diriez-vous que vous êtes?

Réponse : pas du tout gêné, peu gêné, Assez gêné, très gêné

A quelle fréquence utilisez-vous les lotions, sprays et crèmes répulsives?

Réponse : Jamais, rarement, de temps en temps, souvent

A quelle fréquence utilisez-vous les diffuseurs insecticides, serpentins, pièges à moustiques?

Réponse : Jamais, rarement, de temps en temps, souvent

A quelle fréquence utilisez-vous les moustiquaires autour du lit ou aux fenêtres?

Réponse : Jamais, rarement, de temps en temps, souvent

A quelle fréquence utilisez-vous le port des vêtements amples?

Réponse : Jamais, rarement, de temps en temps, souvent

A quelle fréquence utilisez-vous la climatisation ou le ventilateur?

Réponse : Jamais, rarement, de temps en temps, souvent

Trouvez-vous qu'il est efficace d'éliminer ou de couvrir les eaux stagnantes pour lutter contre la prolifération des moustiques chez vous et autour de chez vous?

Réponse : pas du tout, Plutôt pas, plutôt, très

Pensez-vous que le Chikungunya peut être transmis ou non par les piqures de moustiques auxquels vous êtes exposés chez vous et autour de chez vous?

Réponse : oui, non

Pensez-vous que le Paludisme peut être transmis ou non par les piqures de moustiques auxquels vous êtes exposés chez vous et autour de chez vous?

Réponse : oui, non

Pensez-vous que la Dengue peut être transmise ou non par les piqures de moustiques auxquels vous êtes exposées chez vous et autour de chez vous?

Réponse : oui, non

Concernant le Chikungunya, diriez-vous que c'est une maladie

Réponse: Pas grave ; peu grave, assez grave, Très grave,

Concernant la Dengue, diriez-vous que c'est une maladie

Réponse: Pas grave ; peu grave, assez grave, Très grave,

L'idée d'attraper une maladie transmise par les moustiques vous inquiète-t-elle?

Réponse : pas du tout, Plutôt pas, plutôt, très

Considérez vous que vous êtes bien informé(e) sur les maladies transmises par les moustiques ?

Réponse : pas du tout, Plutôt pas, plutôt, très
