# Supplementary material for: Inferring human behavior through online social networks may provide accurate behavioral estimates for outbreak forecasting of arboviruses
Source: PLOS Glob Public Health. 2025 Jul 24;5(7):e0004889. doi: 10.1371/journal.pgph.0004889 (PMC12289027; doi:10.1371/journal.pgph.0004889)
Supplement: S2 File — (DOCX) [file pgph.0004889.s002.docx]

***Words identified with Nvivo***

| allergie-allergique | endormi-rendormir-dormir | produit |
| --- | --- | --- |
| antimoustique/anti-moustique | entend | prolifération |
| appartement | environnement | raquette |
| attaque | essaye | reproduction |
| attention | exploser | responsable |
| attraper | gratte-gratter | réveiller |
| autour | horrible | satisfaction |
| beaucoup | impossible | sensation |
| bouffer | impression | solution |
| bourdonnement | inoffensif | sommeil |
| bouton | insecticide | surveillance |
| bracelet | insupportable | technique |
| bruit | intelligent | tellement |
| campagne | invisible | tigre |
| chaleur | l'heure | tourne |
| chambre | Martinique | tranquille |
| chasse | matin | transmis-transmission |
| cherche | monde | vacances |
| cheville | moustiquaire | vaporisateur |
| chikungunya | moustiquesol | veiller |
| citronnelle | néorésistant | viens |
| commence | neutraliser | vigilance |
| confiance | niquer | violence |
| couette | oreilles-oreille | virus |
| couverture | paisiblement | visage |
| dangereux | paludisme | Zika |
| département | petit |  |
| électronique | piquer-piqure-piqures |  |
